# Implementation of a teen sleep app in Canadian high schools: Preliminary evidence of acceptability, engagement, and capacity for supporting healthy sleep habits

**DOI:** 10.1111/jsr.14199

**Published:** 2024-03-20

**Authors:** Parky H. Lau, Colleen E. Carney

**Affiliations:** ^1^ Department of Psychology Toronto Metropolitan University Toronto Ontario Canada

**Keywords:** adolescent, evidence‐based, high school, sleep health

## Abstract

High school students suffer from mental health challenges and poorer academic performance resulting from sleep disturbances. Unfortunately, approaches to this problem sometimes focus on increasing sleep duration by going to bed early; a strategy with limited success because teens experience a phase delay in bedtimes. There is a need for approaches that leverage behavioural sleep science and are accessible, scalable, and easily disseminated to students. DOZE (Delivering Online Zzz's with Empirical Support) is a self‐management app that is grounded in sleep and circadian basic science. Although initial testing supports it as a feasible and acceptable app in a research context, it has not been tested as a strategy to use in schools. The present study tested DOZE in private high schools in Canada. Two‐hundred and twenty‐three students downloaded the app and completed daily sleep diaries over 4 weeks. Students reported a more regularised routine for bedtime, M_diff_ = −0.43 h, *p* < 0.001, 95% CI [−0.65, −0.21], and rise time, M_diff_ = −0.61 h, *p* < 0.001, 95% CI [−0.84, −0.38], in addition to a higher total sleep time, M_diff_ = 0.18 h, *p* < 0.008, 95% CI [0.05, 0.31]. Students also rated DOZE to be highly acceptable. The evidence suggests that students find DOZE to be acceptable and engagement in this nonclinical population was reasonably high under minimal researcher supervision. This makes DOZE an attractive option and a step towards broad‐based sleep health services. High powered replications with control groups are needed to increase empirical rigour.

## 1 INTRODUCTION

Poor sleep is pervasive among high school students, which has substantial negative effects on academic performance and mental health (Kaneita et al., 2009; Ming et al., 2011). For example, Zhang et al. (2017) conducted a large‐scale nationally representative cross‐sectional survey and found that sleep disruptions were associated with increased odds of mood, anxiety, substance use, and suicidality. Consequently, adolescent sleep is considered a public health crisis and schools are in need of a solution. Unfortunately, some approaches to this problem focus on increasing sleep duration by going to bed early (e.g., Matsumoto et al., 2021; Owens et al., 2006). This is a strategy that confers limited success because adolescents experience a phase delay that limits their ability to go to sleep early and to rise early. Specifically, Carskadon (2011) elaborated that changes in homeostatic and circadian processes drive adolescents towards a later bed time, which is in direct opposition to psychosocial demands to wake up earlier (i.e., school start times). The result is a “perfect storm” of shortened and ill‐timed sleep in adolescents and young adults.

The ramification of this conflict is evident in the literature. For example, one meta‐analysis (Gradisar et al., 2011) found that more than half of adolescents obtained less than 8 hours of sleep on school nights which is below the recommended sleep duration (Paruthi et al., 2016). This trajectory of shortened sleep has been an insidious development. For example, longitudinal work has elucidated a progressive reduction in sleep duration in Canadian adolescents over three waves (Patte et al., 2017). In response to this shortened sleep, adolescents may compensate for the accruing sleep debt by sleeping in on weekends. However, weekday–weekend discrepancies in bed/rise times contribute to significant sleep variability that can lead to feelings of discomfort and poor timing of sleep and wake (also known as “social jetlag”; Wittmann et al., 2006).

Some possible reasons for the increasingly prevalent sleep difficulties in this population could be related to the increase in sedentary behaviours because of phone and computer use (Bassett et al., 2015; Yang et al., 2019). These lifestyle changes may be reducing the overall build‐up of homeostatic sleep pressure, which can exacerbate delayed bed times and reduce the overall quality of sleep in adolescents. To compensate in the day, teens may pull for rest and reduced activity which contributes to more sleep disturbances. There is also research to suggest that evening light exposure related to screen use can play a role in reducing sleep quality and bed timing (Chinoy et al., 2018; Hartley et al., 2022). One review on the relationship between screen time and sleep in adolescents by Hale and Guan (2015) found consistent significant negative associations between greater evening screen use and reduced total sleep time.

Besides sleep, we also see general trajectories of increased anxiety and mood difficulties among adolescents (Parodi et al., 2024; Thorisdottir et al., 2017). Higher arousal levels in anxiety are incompatible with sleep. Moreover, loss of motivation in depression may pull for reduced activity and greater rest, consistent with previously discussed points in reduced homeostatic pressure. Consequently, dealing with some of these possible challenges could be one way to add reprieve to the adolescent sleep crisis.

### 1.1 Limitations with current treatment options for adolescent sleep health

Consequently, there is a need for approaches that leverage behavioural sleep science and are accessible, scalable, and easily disseminated to high school students. However, current treatments are limited in scope, tolerability, and/or scalability in addressing the public health crisis of adolescent sleep problems. Pharmacological options, such as medication (benzodiazepines and Z drugs, such as zopiclone) come at the cost of significant side effects and do not treat the symptoms at its cause. Over the counter supplements, such melatonin, are not strictly FDA regulated as they are considered a dietary supplement and are susceptible to misuse (Lelak et al., 2022).

With respect to nonpharmacological evidence‐based treatments of sleep disorders, the gold standard for the treatment of insomnia – a common sleep problem in adolescents (Johnson et al., 2006) – is cognitive behavioural therapy. CBT for insomnia has been found to be an effective intervention in adolescents (e.g., Ma et al., 2018). However, one limitation of CBT for insomnia is in its namesake. Adolescents commonly present with other sleep challenges such as chronic sleep deprivation and circadian misalignment. Consequently, CBT for insomnia may be too specific a treatment to support multifaceted sleep health in adolescents.

Beyond CBT, there are sleep treatments that have been developed using a transdiagnostic framework to address the multitude of sleep problems that may present in this population. For example, the Transdiagnostic Intervention for Sleep and Circadian Dysfunction (TranS‐C; Harvey, 2016) is a modular treatment that targets different common issues of sleep. Evidence suggests that TranS‐C is an important step towards fostering comprehensive sleep health and the treatment has been found to increase the total sleep time and a shift towards morningness in adolescents (Dong et al., 2019; Gasperetti et al., 2022). Similar to CBT‐I, TranS‐C is typically clinician‐led, which reduces accessibility. Moreover, the treatment may be too intensive for youths who simply want additional support with sleep but do prefer not to commit to a specific clinician‐led treatment protocol.

### 1.2 A web‐based app for sleep in adolescents

In response to the paucity of evidence‐based treatments that address different areas of sleep concerns, are accessible, and cater to the adolescent and young adult population, we spearheaded a project to develop DOZE (Delivering Online Zzz's with Empirical Support). DOZE is a behavioural, self‐management, sleep app that was co‐designed through an iterative process based on feedback from both adolescents and young adults (AYAs) and healthcare provider stakeholders to ensure that the app is tailored specifically to AYA needs (Hackett et al., 2018; Thabrew et al., 2018). For example, initial design consultations revealed that AYAs preferred an app that: (1) included a sleep diary for self‐monitoring; (2) provided feedback on their personal sleep habits; (3) opportunities to set personal sleep goals; and (4) expert‐level recommendations to make changes to their sleep based on these goals. Following this initial phase, a user‐informed redesign of DOZE was conducted with an industry partner (PIVOT Design), consistent with standard app development processes (Shah et al., 2019), including six user assessments with AYAs (aged 15–24 years) on iterative prototype versions of the app and consultations with health care provider stakeholders. A more fulsome description of what the intervention entails is included in the Methods section.

With respect to initial evidence for feasibility and acceptability of DOZE, Carmona et al. (2021) studied 83 adolescents and young adults and their use of DOZE over 4 weeks. Participants reported significant improvements in prospective sleep indices (e.g., lingering in bed, total wake time, total sleep time, sleep efficiency), and self‐reported subjective insomnia severity. Moreover, participants also improved in their reports of anxiety, depression, and fatigue. In terms of acceptability, DOZE was rated as an acceptable and credible intervention.

## 2 THE PRESENT STUDY

Current evidence for DOZE suggests that it is an accessible and tolerable intervention within a research context. However, empirical endeavours to validate DOZE lack ecological validity: participants were recruited because they expressed dissatisfaction with their sleep and received compensation for engaging in the study. The central goal of the DOZE project is to provide to the community at large an accessible option that is backed by sleep and circadian basic science and to be part of a broader solution to the public sleep health crisis in adolescents. In the spirit of implementation science, we need to move closer towards the effectiveness side of the efficacy–effectiveness continuum. Therefore, we need to know whether disseminating and implementing DOZE into the community (e.g., high schools) can be effective and well tolerated even in the absence of significant researcher or clinician involvement.

The present study introduced DOZE to private high schools in Canada. After proposing the study to relevant stakeholders (i.e., social workers and teachers), DOZE was disseminated to students in order to evaluate the levels of engagement, acceptability, and effectiveness of the intervention. Should DOZE be found to be a useful solution towards improving sleep health in this context, then this may have implications towards greater widespread access to evidence‐based sleep interventions in the community.

## 3 METHODS

### 3.1 Participants

Participants included adolescents aged 13 to 17 at private high schools in British Columbia and Ontario, Canada. Besides age range, there were no other exclusion criteria. We were interested in understanding how DOZE can be implemented into the school community, so all students had access to the intervention regardless of the presence or absence of sleep difficulties. This approach goes beyond removing sleep pathology and moves towards fostering sleep health.

### 3.2 Intervention

DOZE is a self‐management web‐based app for sleep disturbances. In the initial onboarding phase, participants were instructed to complete the sleep diary every morning and input it directly into the app. DOZE provided personalised feedback after 2 weeks on sleep indices. Specifically, the app indicated whether sleep indices are normal, too high, or too low on the Progress screen. Users then had the opportunity to set goals to improve their sleep in problem areas. These feedback areas represent components of evidence‐based treatment (Harvey & Buysse, 2017) and included: naps, excessive/too little TIB, lingering in bed in the morning, sleepiness, and irregular schedule. These parameters were based on age‐adjusted norms presented in Hysing et al. (2013). For example, adolescents are typically recommended to sleep between 8 to 10 h per night (Paruthi et al., 2016). Consequently, we operationalised excessive time in bed as spending over 11 h in bed and too little time in bed as below 8 h. For sleep efficiency, we used typical norms in CBT‐I, with efficiencies between 85% and 90% viewed as within normal limits.

Students also had the opportunity to set personalised goals in these areas, such as reducing excessive time spent in bed or increasing sleep duration. However, they would not be allowed to set goals if their personalised data suggested that they were already within normal limits in an area. For example, a student would not be asked to set a goal to sleep more if their sleep efficiency was within normal limits and sleep duration was between 8 and 10 h.

In addition, participants could access a Tips section that included quizzes and psychoeducation about hyperarousal (feeling on high alert at night), chronotype (morning birds vs night owl), difficulty getting out of bed in the morning, daytime sleepiness (trouble staying awake during the day), and fatigue (e.g., tips on how to have more energy throughout the day, such as proper hydration, balanced meals, increasing activity, and getting sunlight exposure). There were also tips to wind‐down and reduce the use of electronics at night. To manage hyperarousal, there was a Relaxation section that provided common relaxation techniques, such as progressive muscle relaxation and guided breathing.

After setting goals, participants then completed sleep diaries for an additional 2 weeks to follow these recommendations and to track progress. Screenshots of the example progress, goal‐setting, and tips screens can be found in Carmona et al. (2021).

### 3.3 Measures

#### 3.3.1 Participant characteristics

A researcher‐developed questionnaire that included age, sex, ethnicity, and educational level was used to characterise the sample. We also included two questions that asked for the average weekly duration that students commit towards academic‐related activities and extracurricular activities.

#### 3.3.2 Feasibility, acceptability, and engagement

##### Acceptability E‐scale

This measure assessed students’ attitudes regarding ease of use, understandability, enjoyment, perceived helpfulness, time commitment, and overall satisfaction regarding DOZE. Participants rated each item on a scale of 1 to 5, with higher scores indicating greater acceptability and more favourable attitudes towards DOZE.

##### Therapy evaluation questionnaire (TEQ; Borkovec & Nau, 1972)

A modified TEQ will be used to evaluate perceived credibility of DOZE. The TEQ has been used in previous clinical trials of sleep medicine treatments (e.g., Carney et al., 2017). Each item is scored from 1 (*not at all*) to 7 (*very*), with higher scores reflecting greater perceived credibility and satisfaction with DOZE. The TEQ has demonstrated acceptable internal consistency (Cronbach's a = 0.79) (Edinger et al., 2001).

##### Engagement

Engagement was defined as a proportion of the number of DOZE accounts created out of students who provided their informed consent. We also looked at sleep diary completion at each of the 2‐week periods (please see Procedures section for an elaboration on the term “period”).

#### 3.3.3 Outcome measures

##### Insomnia severity index (ISI; Morin, 1993)

The ISI is a 7‐item self‐report measure that assesses subjective insomnia symptom severity, satisfaction, distress, and impairment with respect to insomnia symptoms. The ISI has demonstrated excellent internal consistency in community and clinical samples (α = 0.90; Morin et al., 2011) and has also been validated in adolescent and teenage samples (Gerber et al., 2016).

##### Fatigue severity scale (FSS; Krupp et al., 1989)

The FSS is a 9‐item scale measuring impairment related to fatigue, and items are rated on a Likert scale from 1 (*no impairment*) to 7 (*severe impairment*). A mean item score is calculated, with higher scores indicating more severe fatigue. The FSS has demonstrated convergent validity with a visual analogue scale for fatigue (Krupp et al., 1989) and has previously been used to measure fatigue in clinical samples of adolescents (Wang et al., 2013).

##### Cleveland adolescent sleepiness questionnaire (CASQ; Spilsbury et al., 2007)

The CASQ which comprises 16 items scored on a 5‐point Likert scale ranging from *never* to *almost every day*. Items are scored to yield a total score. The CASQ has been validated in samples of clinical and nonclinical adolescents with high internal consistency (Cronbach's a = 0.89) and strong convergent validity with two other measures of daytime sleepiness (Spilsbury et al., 2007).

##### State–trait inventory of cognitive and somatic anxiety (STICSA; Ree et al., 2008)

The STICSA comprises scales assessing the presence of cognitive and somatic symptoms of anxiety when the measure is being completed (State) and anxiety in general (Trait). The STICSA has demonstrated acceptable internal consistency (Cronbach's a = 0.77 for the Cognitive subscale and 0.74 for the Somatic subscale) (Deacy et al., 2016).

##### The center for epidemiologic studies depression scale – revised 10‐item version for adolescents (CESD‐R‐10; Radloff, 1977)

This was included to evaluate depression symptom severity. Participants rated the frequency of each symptom during the past 2 weeks, ranging from 0 (*not at all or less than 1 day*) to 4 (*nearly every day for 2 weeks*). The CESDR‐10 has demonstrated excellent internal consistency in adolescents (McDonald's omega = 0.87) (Ashaie & Cherney, 2021).

##### 13‐Item composite scale of morningness (CSM; Smith et al., 1989)

The CSM, which refers to one's endogenous circadian preference (i.e., morning‐bird, night‐owl, or somewhere in between), was measured using the 13‐item Composite Scale of Morningness. Higher scores represent greater tendencies towards morningness. The CSM has been validated in samples of adolescents and undergraduate students with good internal consistency (Cronbach's a = 0.86 for adolescence and 0.84 in undergraduates) (Jankowski, 2015; Randler, 2008).

##### 36‐item short form survey instrument (SF‐36; Ware Jr & Sherbourne, 1992)

The SF‐36 consists of 36 items that assess various domains of functioning. Subscales have demonstrated good convergent and discriminant and validity (Lin et al., 2020) in adolescent samples with high Cronbach's alpha (range 0.82–0.91) (Gee et al., 2002).

##### Consensus sleep diary (CSD; Carney et al., 2012)

DOZE included an adaptation of the Consensus Sleep Diary (CSD; Carney et al., 2012), which assesses sleep habits during the 24 h period: bedtime, sleep attempt, sleep onset latency (SOL; i.e., how long it took to fall asleep); wake after sleep onset (WASO; i.e., length of awakenings after falling asleep); final awakening, rise time (i.e., time of getting out of bed), and naps. These responses will be used to calculate additional sleep indices, such as average bed and rise time, variability in rise time (range of earliest to latest rise time), variability in bedtime (range of earliest to latest bedtime), morning lingering in bed (the difference between time of final awakening and rise time), total sleep time (TST; total time spent asleep), total wake time (TWT; total time spent awake during the sleep period), time in bed (TIB; bedtime to rise time), and sleep efficiency (SE; percent of TIB spent asleep). Total sleep time and average bed/rise times for weekdays and weekends were also calculated by the researcher, given that greater weekday–weekend sleep discrepancies can increase social jetlag symptoms and reduce sleep quality. Participants were able to view their average sleep indices over a 2 week period on a personalised dashboard.

### 3.4 Procedures

**FIGURE 1:**
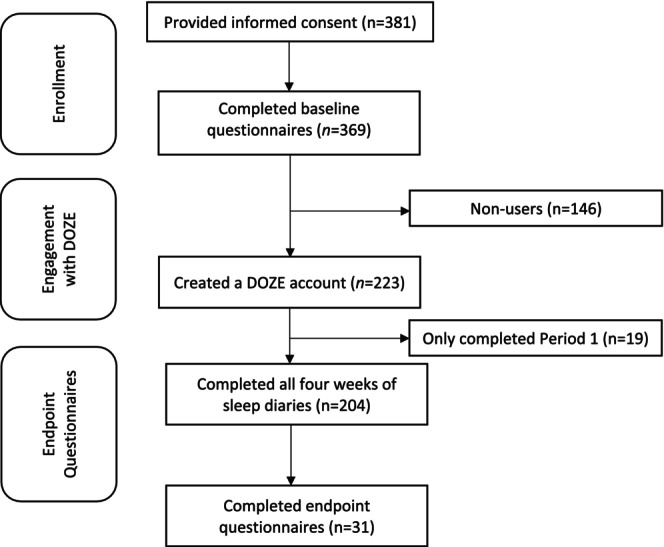
Flowchart of participants.

The researcher initiated contact with stakeholders (i.e., social workers, teachers) from private high schools in British Columbia and Ontario, Canada to garner interest in participating in DOZE. The first author then provided a detailed overview of the DOZE project with stakeholders and then elicited feedback about possibilities for collaboration. After receiving consent from the school, the study was then disseminated to students. The specific dissemination strategy differed based on the school from the use of a newsletter to implementing the programme within the physical education curriculum. Teachers or social workers supported students in providing the students with the links to the consent form (all participants provided their informed consent), which then allowed students to access the baseline questionnaires. Students were then directed to download the app and link their research ID to their DOZE accounts. Students completed an initial 14 days of sleep diary observations on the DOZE app. This observational data gathering period is Period 1. Afterwards, they were given a chance to set sleep goals (e.g., increase sleep time, spend less time in bed awake, increase energy) and they were provided expert‐level recommendations by the app based on the sleep data collected. Students then completed an additional 14 days following recommendations (Period 2) on DOZE to assess changes in sleep patterns. After these 4 weeks, students completed an endpoint questionnaire that was sent in a separate email. This study received ethics approval from both the schools and the university (REB2022/054). This study was not pre‐registered, though the initial study described in Carmona et al. (2021) was pre‐registered (https://clinicaltrials.gov/study/NCT03960294). For a flowchart of participants, see Figure 1.

### 3.5 Statistical analyses

The function of the analyses was primarily to evaluate acceptability, credibility, engagement, and effectiveness of DOZE in high school students. Descriptive statistics were used to provide sample characteristics in terms of sociodemographic variables. Additionally, independent samples pairwise comparisons and chi‐square tests were employed to determine whether users differed on any of the sociodemographic or clinical variables from non‐users (i.e., those who did not create a DOZE account). Pairwise *t*‐tests were employed to evaluate the effectiveness of DOZE on sleep and other health related outcomes. These analyses were bootstrapped with 1000 samples to account for slight deviations from normality. Effect sizes (Cohen's *d*) were provided for these analyses and follow original guidelines for interpreting small, medium, and large effects (Cohen, 2016). Sleep diary indices were analysed if at least seven nights were completed at each time point because the literature suggests that is the minimum number to reliably estimate adolescent sleep (Short et al., 2017). Finally, a combination of descriptive statistics and inferential statistics using pairwise comparisons were used to evaluate engagement, acceptability, and credibility.

## 4 RESULTS

### 4.1 Study participants

A total of 369 students aged 13 to 17 participated in the study. The sample was primarily White males between Grade 9 to 11. A full breakdown of participant demographics can be found in Table 1.

**TABLE 1:** Participant characteristics for the total sample.

Variable	Mean (SD) or count (%)
Age	14.96 (1.20)
Sex	
Female	25 (6.8%)
Male	344 (93.2%)
Ethnicity	
African	12 (3.3%)
Caribbean	7 (1.9%)
East/Southeast Asian	41 (11.1%)
European	226 (61.2%)
Latin/Central/South American	6 (1.6%)
Oceanic	2 (0.5%)
South Asian	13 (3.5%)
West Asian/Arab	5 (1.4%)
Other/Prefer not to answer	53 (14.3%)
School Grade	
Grade 8	5 (1.4%)
Grade 9	136 (36.9%)
Grade 10	120 (32.5%)
Grade 11	102 (27.6%)
Grade 12	6 (1.6%)
Weekly Academic Commitment	10.72 h (8.70)
Weekly Extracurricular Commitment	10.41 h (5.60)

### 4.2 Characteristic differences between users and non‐users

In terms of sociodemographic characteristics, between‐groups tests found statistically significant differences in age and sex. Users were more likely to be older (*t*[365] = 2.72, *p* = 0.007) and male (χ^2^[2] = 16.51, *p* < 0.001) compared with their non‐user counterparts. Sex differences are likely because most students participating were from an all‐boys private high school. Users also had lower scores on the cognitive component measured by the STICSA, (*t*[355] = 2.86, *p* = 0.007), but not the somatic component (*p* = 0.07). Finally, users reported fewer weekly hours of academic commitment compared with non‐users (*t*[359] = −2.51, *p* = 0.013). No other statistically significant differences were found.

### 4.3 Engagement with DOZE

There were 369 students who completed the consent form and baseline questionnaires. Of this initial sample, 223 students created a DOZE account. Consequently, the engagement rate based on this definition was 60.4%. Mean diary completion for the first period was 11.96 (SD = 2.51) and second period was 8.74 (SD = 4.92). Moreover, binary (yes/no) definition of sleep diary completion was based on previous literature indicating that the minimum number of sleep diaries to reliably estimate adolescent sleep (≥7 days; Short et al., 2017). Completion rates at Period 1 and Period 2 were 99.6% (*n* = 222/223) and 73.1% (*n* = 163/223), respectively. This suggests that almost all students completed the observational period and a significant majority continued in Period 2 where feedback was provided.

### 4.4 Changes in prospective sleep diary indices after DOZE

Paired samples *t*‐tests determined statistically significant changes in bed and rise time variability, total sleep time, total wake TIB, with increased SE. Specifically, adolescents’ sleep patterns were more regularised in terms of bed time, M_diff_ = −0.43 h, *p* < 0.001, 95% CI [−0.65, −0.21], and rise time M_diff_ = −0.61 h, *p* < 0.001, 95% CI [−0.84, −0.38]. Moreover, they reported higher sleep duration, M_diff_ = 0.18 h, *p* < 0.008, 95% CI [0.05, 0.31] and spent less time awake in bed, M_diff_ = −0.05 h, *p* = 0.03, 95% CI [−0.10, 0.00]. Overall, the total time in bed increased because of the higher sleep duration, M_diff_ = −0.17 h, *p* = 0.03, 95% CI [0.01, 0.32] and sleep efficiency also increased, M_diff_ = 0.02, *p* < 0.001, 95% CI [0.00, 0.03]. No other findings were statistically significant (*p*s >0.14). For a detailed summary of sleep diary related changes, see Table 2.

**TABLE 2:** Means (SD) of prospective sleep diary indices at Period 1 and Period 2 with calculated effect sizes and *t*‐statistics.

Measure	Period 1, mean (SD)	Period 2, mean (SD)	Cohen's *d*	*t*(df), *p* value
Bed time	23:07 (0:47)	23:05 (0:47)	0.04	*t*(203) = 0.52, *p* = 0.60
Weekday	23:03 (0:47)	23:01 (23:01)	0.06	*t*(195) = 0.80, *p* = 0.42
Weekend	23:14 (0:59)	23:14 (0:58)	0.01	*t*(184) = −0.13, *p* = 0.89
Rise time	7:45 (0:35)	7:56 (0:49)	0.11	*t*(203) = −1.45, *p* = 0.15
Weekday	7:44 (0:38)	7:47 (0:49)	−0.09	*t*(195) = −1.21, *p* = 0.23
Weekend	8:10 (0:56)	8:12 (1:07)	−0.02	*t*(184) = −0.29, *p* = 0.78
Bed time variability*	2:59 (1:24)	2:34 (1:17)	0.29	*t*(203) = 3.82, *p* < 0.001
Rise time variability*	4:03 (1:31)	3:26 (1:37)	0.39	*t*(203) = 5.20, *p* < 0.001
Napping duration	0:14 (0:36)	0:11 (0:03)	0.05	*t*(203) = 0.72, *p* = 0.47
Total sleep time*	7:50 (0:52)	8:01 (1:05)	−0.20	*t*(203) = −2.70, *p* < 0.01
Weekday*	7:49 (0:53)	7:59 (0:55)	−0.24	*t*(195) = −3.18, *p* < 0.01
Weekend*	7:56 (1:05)	8:10 (1:06)	−0.22	*t*(184) = −2.86, *p* < 0.01
Total wake time*	0:31 (0:20)	0:28 (0:24)	0.16	*t*(203) = 2.14, *p* = 0.03
Morning lingering in bed	0:11 (0:08)	0:11 (0:12)	−0.03	*t*(203) = −0.46, *p* = 0.65
Time in bed*	8:18 (1:02)	8:28 (0:05)	−0.16	*t*(203) = −2.14, *p* = 0.03
Sleep efficiency*	0.94 (0.06)	0.95 (0.10)	−0.19	*t*(203) = −2.47, *p* = 0.02

*Note*: Sleep indices are presented in hh:mm format.

* *p* < 0.05.

### 4.5 Effectiveness of DOZE on retrospective outcomes

Response rates for completed endpoint questionnaires were low (*n* = 31). However, a statistically significant difference was found for ISI scores, M_diff_ = −1.44, *p* = 0.015, 95% CI [−2.59, −0.30]. There were no other statistically significant differences in outcome measures in terms of self‐reported sleepiness, chronotype, fatigue, depression, anxiety, or general health and wellbeing (*p*s >0.05). For a summary, see Table 3.

**TABLE 3:** Means (SD) of retrospective outcome measures at Period 1 and Period 2 with calculated effect sizes and *t*‐statistics.

Measure	Period 1, mean (SD)	Period 2, mean (SD)	Cohen's *d*	*t*(df), *p* value
ISI*	8.41 (4.05)	6.96 (4.19)	0.50	*t*(26) = 2.60, *p* = 0.015
FSS	3.57 (0.79)	3.52 (1.04)	0.05	*t*(27) = 0.28, *p* = 0.78
CSM	32.50 (6.81)	32.46 (6.81)	0.01	*t*(25) = 0.07, *p* = 0.95
CASQ	30.05 (5.24)	37.82 (6.15)	0.04	*t*(21) = 0.20, *p* = 0.84
STICSA Cognitive	16.79 (4.46)	16.07 (5.29)	0.32	*t*(27) = −1.21, *p* = 0.10
STICSA Somatic	16.07 (3.73)	15.11 (2.92)	0.18	*t*(27) = 0.95, *p* = 0.35
CESD‐R	7.96 (6.44)	8.21 (6.90)	−0.06	*t*(23) = −0.27, *p* < 0.79
SF36	73.27 (16.91)	74.42 (16.57)	−0.08	*t*(25) = −0.41, *p* < 0.69

Abbreviations: CASQ, Cleveland Adolescent Sleepiness Questionnaire; CESD‐R, The Center for Epidemiologic Studies Depression Scale–Revised; CSM, 13‐Item Composite Scale of Morningness; FSS, Fatigue Severity Scale; ISI, Insomnia Severity Index; SF36, 36‐Item Short Form Survey Instrument; STICSA, State – Trait Inventory of Cognitive and Somatic Anxiety.

* *p* < 0.05.

### 4.6 Evidence of acceptability and credibility

With regard to the Acceptability E‐Scale, modal analyses suggested that overall satisfaction with the app was 4 (out of a possible score of 5). In particular, students found DOZE to be acceptable in terms of time efficiency (5), being understandable (5), and easy to use (5). DOZE was also seen as generally acceptable in terms of enjoyment (3) and helpful for sleep disturbances (3). General TEQ scores remained quite stable, with a marginally statistically significant increase (*p* = 0.06). Exploratory pairwise comparisons at the item level found an increase in how much students would recommend DOZE to a friend to support sleep health, M_diff_ = 0.68, *p* = 0.01, 95% CI [0.16, 1.20].

## 5 DISCUSSION

The present study implemented and disseminated DOZE into private high schools in Canada to offer a low cost, flexible, and scalable sleep health option to adolescents. Overall, DOZE was observed as an acceptable intervention and students experienced some improvements for sleep habits related to regularity and duration. These small to medium improvements positively contribute to two key areas of concern in adolescents: chronic sleep deprivation and social jetlag from irregular sleep/wake patterns. Moreover, pre–post tests also elucidated an improvement (medium effect size) in retrospective symptoms of insomnia, suggesting an overall reduction in perceptions of sleep disturbance. However, the absolute value change of −1.4 is unlikely clinically relevant, which could be a combination of a floor effect given the healthy teenage sample or a lack of utility of DOZE in reducing perceptions of sleep disturbance.

Beyond improvements in sleep habits, the use of DOZE itself was regarded as a highly acceptable and non‐invasive solution. In particular, students generally rated the app as easy to use, understandable, and time efficient. The personalisable and flexible aspect likely supported higher levels of engagement even in the absence of researcher incentive and even within a younger nonclinical sample. Thus, DOZE holds the dialectic of utility and acceptability in fine fashion, which renders DOZE a potentially useful tool that many adolescents can use without significant barriers to entry. Moreover, personal engagement with the app itself is highly modifiable based on the students’ own goals and ability to commit to their sleep health, given other life priorities. This may paradoxically increase engagement with the app by emphasising individual autonomy (e.g., van Dijk et al., 2023).

Although DOZE is a promising solution to providing an accessible and scalable solution for the adolescent sleep crisis, there are caveats that warrant discussion. First, the improvements observed are generally modest and do not address all presenting sleep concerns. For example, sleep efficiency in the sample remained high (95.5%). In this case, policy recommendations (e.g., delaying school start times) should be considered in concert with behavioural interventions to allow for sufficient sleep opportunity. There is also room for the use of transdiagnostic treatments such as TranS‐C, where clinician support might be particularly beneficial in helping students to address key components of sleep issues that are challenging to modify independently, such as buffering against a phase delay (shifting to an earlier bed/rise time).

This study is only the first preliminary exploration into DOZE as an acceptable and useful tool for high school students. Additional research is needed to address study limitations and to permit grounds for further inquiry. With regard to engagement, rates of downloading the app and sleep diary completion were reasonably high, given the population under study. However, the number of students who participated in the study varied based on how each school implemented DOZE. Schools that actively integrated the app into their physical education class saw higher participation in the study at the school level compared with those who advertised the study in newsletters. Consequently, follow‐up studies with stakeholders (e.g., social workers, teachers) using qualitative interviews need to be conducted in order to co‐discover the best practices to facilitate access and engagement with DOZE.

Additional research also needs to be conducted to address methodological limitations. The sample was primarily male owing to the fact that most participants came from an all‐boys private school. Moreover, the majority of the sample also came from one school. Having a larger sample from various schools participating in the research would allow for mixed modelling that would identify possible moderators of outcome and engagement at levels of both the individual and the school level. Moreover, even though prospective sleep diary completion was high, the absence of compensation reduced the incentive to complete the final set of retrospective outcome measures. The significant loss of sample at this time point hampered statistical power, increasing the risk of type II errors. A past study with DOZE (Carmona et al., 2021) saw improvements in anxiety, depression, and fatigue in that sample. Finally, the lack of a control group weakens internal validity; we cannot conclude whether improvements in sleep‐related habits are related to the app or if other factors (e.g., holidays, exam periods, statistical regression towards the mean through multiple measurements) played a contributing role. Therefore, high powered replications with a control condition are important to include to better assess outcomes. Finally, it would be helpful to evaluate the maintenance of DOZE in terms of use and effectiveness in supporting long‐term behaviour change and sleep health.

## 6 CONCLUSIONS

There is a need for accessible and scalable options to support adolescent sleep health. This preliminary study offers DOZE as a promising step forward towards the broader goal of providing accessible sleep healthcare and applying the science of sleep and circadian health into practice within the community. The accessible, flexible, and non‐invasive nature of an evidence‐informed, web‐based self‐management app for sleep disturbances may be particularly useful for nonclinical samples by reinforcing sleep health in a flexible and acceptable manner. However, studies looking at long‐term effectiveness and moderators of acceptability, engagement, and effectiveness are needed. There is a continued need for collaboration and co‐discovery with stakeholders in the community to identify best practices to support fulsome sleep health in adolescents.

## AUTHOR CONTRIBUTIONS

**Parky H. Lau:** Conceptualization; methodology; formal analysis; data curation; writing – original draft; writing – review and editing. **Colleen E. Carney:** Supervision; writing – original draft; methodology; writing – review and editing; conceptualization; resources.

## CONFLICTS OF INTEREST

The authors declare no competing interests. Dr. Colleen Carney worked in collaboration with TochTech to develop dozeapp.ca. Dr. Carney provides financial support to TochTech to develop and maintain the app. The teen sleep app discussed in this manuscript is free and accessible to all teens that are interested in DOZE.

## Data Availability

The data that support the findings of this study are available on request from the corresponding author. The data are not publicly available due to privacy or ethical restrictions.
